# *lptG* contributes to changes in membrane permeability and the emergence of multidrug hypersusceptibility in a cystic fibrosis isolate of *Pseudomonas aeruginosa*

**DOI:** 10.1002/mbo3.844

**Published:** 2019-04-12

**Authors:** Lucas B. Harrison, Randal C. Fowler, Baha Abdalhamid, Anna Selmecki, Nancy D. Hanson

**Affiliations:** ^1^ Department of Medical Microbiology and Immunology Creighton University Omaha Nebraska; ^2^ Department of Pathology and Microbiology University of Nebraska Medical Center Omaha

**Keywords:** carbapenem resistance, cystic fibrosis, hypersusceptibility, lipopolysaccharides, *lptG*, subinhibitory

## Abstract

**Purpose:**

In the lungs of cystic fibrosis patients, *Pseudomonas aeruginosa* is exposed to a myriad of antibiotics leading to alterations in antibiotic susceptibility. This study identifies mutations resulting in hypersusceptibility in isogenic mutants of a *P. aeruginosa* clinical isolate, PA34.

**Methods:**

PA34 was exposed to subinhibitory concentrations of doripenem or meropenem during growth to mid‐log phase. Antibiotic susceptibility of surviving colonies was determined by agar dilution. Two carbapenem‐resistant colonies hypersusceptible to non‐carbapenem antibiotics were selected for further analysis. Antibiotic resistance gene expression was evaluated by RT‐rtPCR and OprD production by SDS‐PAGE. PA34 and isogenic mutants were evaluated with whole genome sequencing. Sequence variants were confirmed by Sanger sequencing, and cognate genes in eight carbapenem‐resistant clinical isolates hypersusceptible to non‐carbapenem antibiotics were sequenced. Lipopolysaccharide preparations of PA34 and hypersusceptible mutants were evaluated with ProQ‐Emerald stain.

**Results:**

Isogenic mutants showed 4‐ to 8‐fold MIC increase for imipenem, meropenem, and doripenem. However, they were hypersusceptible (≥4‐fold MIC decrease) to aminoglycosides, fluoroquinolones, and non‐carbapenem β‐lactams. Expression of *ampC* or *mex*‐*opr* efflux pumps was unchanged, but OprD production was decreased. Mutations causing Q86H AlgU and G77C LptG amino acid substitutions and nonsense mutations within OprD were observed in both mutants. Lipopolysaccharide modifications were observed between isogenic mutants and PA34. Non‐synonymous mutations in LptF or LptG were observed in 6/8 hypersusceptible clinical isolates resistant to carbapenem antibiotics.

**Conclusion:**

Evaluation of hypersusceptible mutants identified the association between *lptG* and a hypersusceptible phenotype. Modifications in lipopolysaccharide profiles suggests LptG modification interferes with lipopolysaccharide transport and contributes to hypersusceptibility.

## Graphical abstract

This study identifies mutations resulting in hypersusceptibility in isogenic mutants and clinical isolates of *Pseudomonas aeruginosa*. Mutations in the gene encoding the LptG subunit of the LptFG heterodimer were found to alter lipopolysaccharide (LPS) profiles in isogenic mutants. Modifications in LPS profiles suggest LptG modification interferes with LPS transport and contributes to hypersusceptibility.

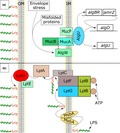

## 1 INTRODUCTION

*Pseudomonas aeruginosa* infections can emerge resistant during antibiotic treatment. Comprehensive transposon‐mutagenesis screens have identified key genes involved in antibiotic susceptibility and hypersusceptibility (Alvarez‐Ortega, Wiegand, Olivares, Hancock, & Martinez, 2010; Breidenstein, Khaira, Wiegand, Overhage, & Hancock, 2008; Dotsch et al., 2009; Fernandez et al., 2013; Schurek et al., 2008). Hypersusceptibility is defined as a ≥4‐fold increase in antibiotic susceptibility and has been observed predominantly in laboratory‐derived mutants of *P. aeruginosa* (Lister, Wolter, & Hanson, 2009; Masuda et al., 1999,2000; Morita, Kimura, Mima, Mizushima, & Tsuchiya, 2001; Morita, Komori, et al., 2001). Although the hypersusceptible phenotype has been linked to alterations in pathways associated with transport and biosynthesis of outer membrane components in clinical isolates of *P. aeruginosa *Angus, Carey, Caron, Kropinski, & Hancock, 1982; Kropinski, Kuzio, Angus, & Hancock, 1982; Ramos‐Aires, Plesiat, Kocjancic‐Curty, & Kohler, 2004), phenotypic induction of hypersusceptibility has only been shown using mutagenic laboratory techniques such as random transposon mutagenesis. The mechanisms by which *P. aeruginosa* develops hypersusceptibility in response to environmental conditions, such as antibiotic exposure, remain unknown.

*Pseudomonas aeruginosa* can survive antibiotic exposure through multiple mechanisms, including biofilm production, the presence of a relatively impermeable outer membrane, regulation of outer membrane porins, antibiotic‐hydrolyzing enzymes, and efflux pumps capable of expelling antibiotics (Yoshimura and Nikaido, 1982). Biofilm production, mediated by multiple factors including AlgU, provides a barrier that inhibits accessibility of antibiotic to the organism. In the outer membrane, antibiotic uptake is inhibited by both membrane‐bound porins and lipopolysaccharide (LPS) composition, as mediated by OprD and the Lpt LPS transport pathway (Fowler & Hanson, 2014; Nikaido & Hancock, 1986; Wolter, Hanson, & Lister, 2004). In the periplasm, an overproduced AmpC cephalosporinase can inactivate a broad range of β‐lactam antibiotics while the Mex‐Opr family of RND efflux pumps removes antibiotics from the periplasm, inner membrane, and cytosol (Wolter, Black, Lister, & Hanson, 2009). The carbapenems (doripenem, meropenem, and imipenem) in addition to colistin are considered last resort antibiotics for the treatment of *P. aeruginosa* infections (Manohar, Babu, Bozdogan, & Ramesh, 2018; Wi et al., 2017). However, mutations that result in truncations in OprD have been associated with imipenem resistance, and when coupled with increased expression of genes encoding the MexAB‐OprM efflux pump lead to decreased susceptibility to meropenem and doripenem (Wolter et al., 2009).

Exposure to subinhibitory concentrations of antibiotics has been shown to select for decreased susceptibility to multiple classes of antibiotics (Kumari, Balasubramanian, Zincke, & Mathee, 2014). In this study, we identified a panel of *P. aeruginosa* clinical isolates showing resistance to doripenem, meropenem, and imipenem, but susceptible to non‐carbapenem antibiotics. We also evaluated how laboratory exposure to subinhibitory concentrations of doripenem or meropenem resulted in the emergence of hypersusceptibility to non‐carbapenem antibiotics concurrent with reduced susceptibility to doripenem and meropenem in a cystic fibrosis isolate of *P. aeruginosa.* To our knowledge, this is the first report of carbapenem exposure resulting in a hypersusceptible phenotype to multiple classes of non‐carbapenem antibiotics in *P. aeruginosa*. The purpose of this study was to use phenotypic, molecular and genomic data to characterize mutations that lead to a hypersusceptible phenotype in isogenic mutants of a *P. aeruginosa* clinical isolate collected from a patient suffering from cystic fibrosis.

## 2 MATERIALS AND METHODS

### 2.1 Antimicrobial susceptibility testing

β‐lactam, aminoglycoside, and fluoroquinolone susceptibilities were determined by agar dilution according to the CLSI guidelines (Clinical & Laboratory Standards Institute, 2012). Disk diffusion tests using rifampicin were performed according to the CLSI guidelines (Clinical & Laboratory Standards Institute, 2012).

### 2.2 Generation of isogenic mutants

Isogenic mutants were generated from a *P. aeruginosa* clinical isolate (PA34) collected from a patient with cystic fibrosis through exposure to subinhibitory concentrations of meropenem and doripenem (Harrison & Hanson, 2017). Briefly, PA34 was challenged during log‐phase growth with either meropenem or doripenem at concentrations equal to 1/2 the MIC as previously described (Fowler, 2014; Thomson, Sanders, & Hayden, 1991). The bacteria were pelleted and resuspended in saline before being inoculated into molten Mueller–Hinton agar containing doripenem or meropenem at the MIC to select for resistant mutants and incubated for 24–48 hr at 37°C. Surviving colonies were subcultured onto blood agar plates and incubated for 24 hr at 37°C before MICs of the isogenic mutants were determined. A 4‐fold increase in MIC to doripenem or meropenem defined each mutant colony and two mutants were selected for further study: PA34‐812M and PA34‐822D. The laboratory strain, PAO1 and a susceptible strain collected from a cystic fibrosis patient, PA443, were used as controls.

### 2.3 Pulsed‐field electrophoresis

Genetic relatedness of the hypersusceptible isogenic mutants was confirmed by pulsed‐field gel electrophoresis (PFGE) analyses using *Spe*I digestion according to the CDC PulseNet protocol with modifications (Goering, Ribot, & Gerner‐Smidt, 2011; Swaminathan, Barrett, Hunter, & Tauxe, 2001). These modifications included culturing *P. aeruginosa* on blood agar plates and in brain‐heart infusion broth, increasing wash duration of post in situ lysis agarose plugs to four cycles of 30 min each and the addition of thiourea (50 µg/ml) in the running buffer during electrophoretic separation of chromosome fragments (Fowler, 2014; Goering et al., 2011; Swaminathan et al., 2001). Eight clinical isolates of *P. aeruginosa* were collected from the King Fahad Specialist Hospital‐Dammam, Saudi Arabia, and susceptibility profiles determined by VITEK2.

### 2.4 Outer membrane protein analysis

Outer membranes were harvested and visualized as previously described (Fowler, 2014; Masuda, Gotoh, Ohya, & Nishino, 1996).

### 2.5 Whole genome sequencing

DNA was harvested from overnight cultures of PA34, PA34‐822D, and PA34‐812M grown in MHB at 37° using the Qiagen Blood and Tissue DNA extraction kit and purified in an Amicon 0.5 ml filter prior to sequencing. Whole genome libraries were prepared using the NexteraXT kit (Illumina) according to the manufacturer's instructions and paired‐end reads (2 × 300) were sequenced on an Illumina MiSeq (Creighton University). Initial de novo assembly of the *P. aeruginosa* genomes was performed using the SPAdes 3.10.1 small genome assembler (Bankevich et al., 2012). Post‐assembly scaffolding and gap closure were performed using RAGOUT, ICORN2, and AlignGraph while PROKKA was used to annotate the finished assemblies (Bao, Jiang, & Girke, 2014; Kolmogorov, Raney, Paten, & Pham, 2014; Otto, Sanders, Berriman, & Newbold, 2010; Seemann, 2014). Chromosomal rearrangements were evaluated using MAUVE (Darling, Mau, Blattner, & Perna, 2004). Single‐nucleotide polymorphisms (SNPs) were identified by mapping reads from the hypersusceptible isogenic mutants of PA34 back to the parent isolate using the bowtie2 (v2.2.6) assembler and the Genome Assembly Toolkit (Langmead & Salzberg, 2012). SNPs were evaluated using both the variant call file metrics and through visual inspection of mapped reads using IGV (Thorvaldsdottir, Robinson, & Mesirov, 2013). Mutations identified were confirmed using Sanger sequencing. The PA34 draft genome is available at DDBJ/ENA/GenBank under the accession PDLR00000000 (Harrison & Hanson, 2017).

### 2.6 Sanger sequencing

The sequences of *lptG* and *lptF* amplicons for PA34, the isogenic mutants and panel of hypersusceptible clinical isolates were determined offsite at Functional Biosciences, Inc. (Madison, WI), by Sanger sequencing (BigDye version 3.1, ABI 3730xl). Samples were prepared according to company submission standards, and the nucleotide quality was called at a Phred score greater than 20.

### 2.7 Protein modeling

Models for proteins of unknown structure were generated using the I‐TASSER suite 4.4 (Zhang, 2008). Ten candidate models were generated for proteins of unknown structure and a representative model for each was selected based on similarity to structure of proteins with known function (e.g., RpoE as a model for AlgU). Normal mode analysis was used to evaluate the flexibility of protein structures based on the interaction of amino acid residues and a differential plot of mobility was used to evaluate changes in flexibility between LptG found in PA34 and G77C‐LptG found in the hypersusceptible isogenic mutants (Ma, 2005).

### 2.8 LPS profiles

Lipopolysaccharide was extracted and diluted to 30 ng/µl using a hot phenol method followed by membrane dialysis (Perry, MacLean, Schollaardt, Bryan, & Ho, 1995). Twenty microliters of the LPS extract was separated using a 12% Tris‐Glycine polyacrylamide gel and visualized using Pro‐Q Emerald 300 stain (Invitrogen).

### 2.9 Gene expression

RNA was extracted as described previously (Wolter et al., 2009). Gene expression was evaluated using Qiagen OneStep RT‐PCR Kit and the Qiagen Rotor‐Gene Q 5‐Plex thermocycler. Fold change in gene expression was calculated using the 2^−ΔΔCt^ method with *rpoD* as the endogenous control.

## 3 RESULTS

Isogenic mutants selected from PA34 demonstrated a 4‐ to 8‐fold decrease in susceptibility to the carbapenem antibiotics imipenem, meropenem, and doripenem (Table 1). Surprisingly, these mutants were hypersusceptible to aminoglycosides, fluoroquinolones, and non‐carbapenem β‐lactams. In comparison to the progenitor PA34, representative mutants generated from exposure to doripenem (PA34‐822D) and meropenem (PA34‐812M) were 4‐ to 32‐fold more susceptible to piperacillin, ceftazidime, aztreonam, and cefepime. In addition, these isogenic mutants were 2‐ to 16‐fold more susceptible to tobramycin, gentamicin, and amikacin with a 2‐ to 8‐fold increase in susceptibility to ciprofloxacin and levofloxacin. Despite the differences in susceptibility, PFGE macrorestriction patterns were indistinguishable between PA34, PA34‐822D, and PA34‐812M, indicating that the hypersusceptible isogenic mutants were highly related (data not shown).

**Table 1:** Antibiotic susceptibility of *Pseudomonas aeruginosa* clinical isolates and isogenic mutants

	MIC (µg/ml) of antibiotic^a^											
Strain/isolate	PIP	CAZ	ATM	FEP	IPM	DOR	MER	TOB	GEN	AMK	CIP	LVX
PAO1	4	1	1	8	2	0.5	0.5	0.5	2	4	0.5	1
PA443	4	1	4	1	2	0.25	0.25	0.5	2	4	0.12	0.5
PA34	**256**	**32**	**4**	**16**	**1**	**0.25**	**0.12**	**8**	**32**	**128**	**0.25**	**0.25**
PA34‐822D	8	2	0.25	4	8	2	1	2	8	16	0.06	0.06
PA34‐812M	16	2	0.5	4	8	2	0.5	4	4	8	0.03	0.06
PA34‐8111M	16	2	0.25	4	8	2	1	4	4	8	0.12	0.12

Parent isolate PA34 susceptibility is indicated in bold.

a Abbreviations: PIP, piperacillin; CAZ, ceftazidime; ATM, aztreonam; FEP, cefepime; IPM, imipenem; MER, meropenem; DOR, doripenem; TOB, tobramycin; GEN, gentamicin; AMK, amikacin; CIP, ciprofloxacin; LVX, levofloxacin.

We then characterized known mechanisms of antibiotic resistance in *P. aeruginosa* using RT‐rtPCR, SDS‐PAGE analysis, and Sanger sequencing. Expression of *ampC*, *oprD*, and the *mex*‐*oprD* efflux pump genes was compared between the susceptible *P. aeruginosa* control strains, the parent isolate PA34, and the hypersusceptible isogenic mutants (Table 2). A 3‐fold increase in *ampC* expression was the only difference observed for PA34‐822D relative to PA34. Although no change in *oprD* expression was observed, SDS‐PAGE analysis revealed a decrease in OprD production to below the limit of detection in the outer membranes of PA34‐822D and PA34‐812M (data not shown). Sanger sequencing analysis of the *oprD* gene revealed a base transition from G → A at position 1,244 that created a premature stop codon (Trp → Stop) in PA34‐822D and a deletion‐induced frameshift at bp 965 resulting in a premature stop codon at amino acid position 345 in PA34‐812M. Structurally, these mutations resulted in truncations of OprD upstream of loop 8 in PA34‐822D and loop 7 in PA34‐812M. These truncations in the OprD protein have been associated with decreased carbapenem susceptibility, a phenotype that is reflected in the isogenic mutants PA34‐812M and PA34‐822D (Quale, Bratu, Gupta, & Landman, 2006; Wolter et al., 2004).

**Table 2:** Transcript levels of antibiotic resistance genes for PA34 and hypersusceptible isogenic mutants

Isolate	Fold change in transcript levels						
	*ampC*	*mexA*	*oprM*	*oprD*	*mexE*	*mexC*	*mexX*
PA34	1	1	1	1	1	1	1
PA34‐822D	3.04	1.51	0.87	1.06	1.06	1.38	1.13
PA34‐812M	0.71	1.14	0.8	0.54	1.43	1.01	1.24

Having identified a known mechanism of carbapenem resistance in the isogenic mutants, our next task was to identify the mechanism of non‐carbapenem antibiotic hypersusceptibility. Whole genome sequencing (WGS) was used to identify genetic differences between isogenic mutants and appraise potential mechanisms for the hypersusceptible phenotype. All mutations resulting in amino acid substitutions or truncations are provided in Table 3. In addition to confirming the mutations in *oprD*, WGS revealed SNPs in genes encoding LptG and AlgU at conserved positions in both mutants. These SNPs resulted in a Q86H substitution in the AlgU transcriptional regulator and a G77C substitution in the LptG subunit of the LPS transport complex LptB_2_FG.

**Table 3:** Mutations selected for by exposure to subinhibitory concentrations of carbapenems

Isolate	*oprD* (bp 964, 965)	*oprD* (bp 1,244)	*algU* (bp 529)	*algU* (bp 258)	*lptG* (bp 229)
PA01	CT	G	C	G	C
**PA34**	CT	G	C	C	C
**PA34‐822D**	CT	**A (stop)**	T	**G (Q86H)**	**T (G77C)**
**PA34‐812M**	**C‐(stop)**^a^	G	T	**G (Q86H)**	**T (G77C)**

Note

Nucleotides in bold indicate non‐silent mutations and corresponding amino acid substitution identified in parentheses. Nucleotide positions relative to parent isolate PA34.

a Frameshift event brings premature stop codon into frame 66bp downstream of thymine deletion.

**Figure 1:**
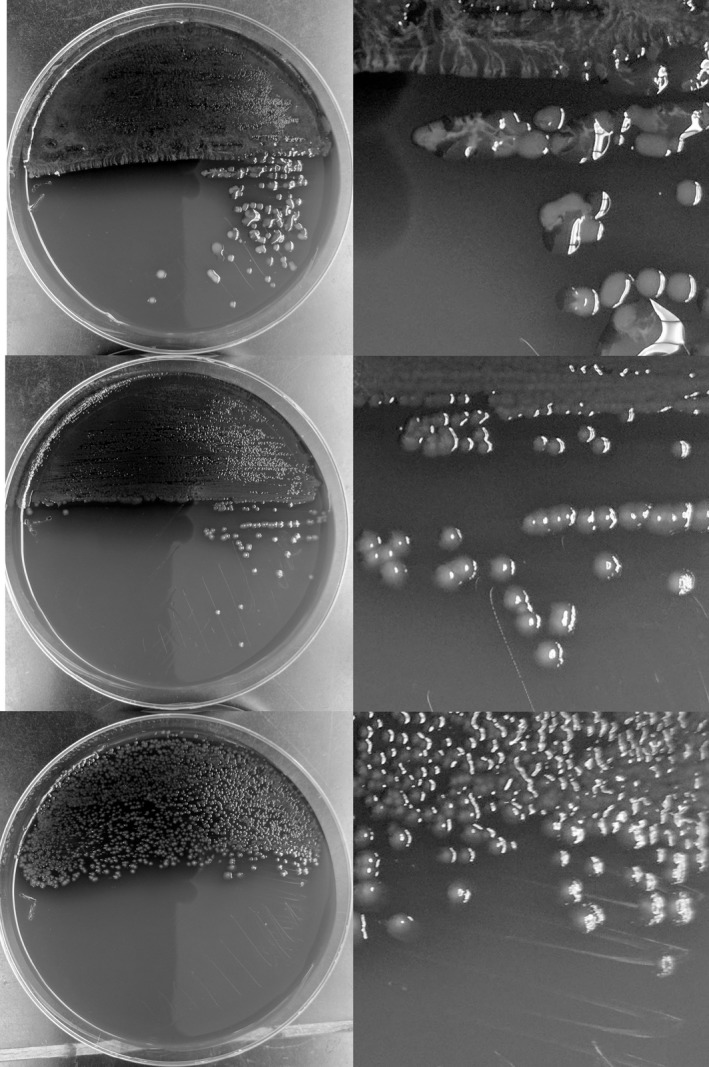
Comparison of colony growth in the mucoid clinical isolate PA34 (top) and the non‐mucoid isogenic mutants PA34‐822D (middle) and PA34‐812M (bottom)

The transcription factor AlgU is responsible for activating a myriad of genes involved in the mucoid phenotype (Firoved & Deretic, 2003). When evaluating mucoid phenotype for PA34 and hypersusceptible isogenic mutants, it was noted that PA34 had a mucoid phenotype but the hypersusceptible isogenic mutants producing AlgU with a Q86H substitution did not (Figure 1). Among the panel of clinical isolates susceptible to non‐carbapenem antibiotics but resistant to doripenem and meropenem, none of the isolates presented a mucoid phenotype.

**Figure 2:**
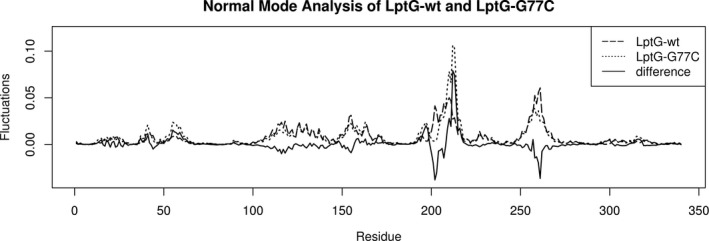
Normal mode analysis informs potential residue displacement at each amino acid position of the protein. Differential analysis of the plots between wild type (wt) (dashed line) and G77C‐LptG (dotted line) show no change in mobility at the substitution site, but the exchange of a nonpolar and flexible glycine for a polar cysteine alters mobility of the other residues as seen at the extra‐membrane sites 200–220 and 255–265

LptG is a subunit of the LptB_2_FG complex that is involved in the transport of LPS from the outer leaflet of the inner membrane to the outer membrane (DeVries & Ohman, 1994). As an initial evaluation of the impact of a G77C substitution in LptG, we compared the structural mobility projections of sequence‐derived models of LptG to the G77C LptG using normal mode analysis. Briefly, normal mode analysis of proteins informs the flexibility of the protein model by evaluating the mobility and environment of each amino acid residue in the structure. Normal mode analysis of a sequence‐derived LptG model protein revealed that the G77C substitution could alter the structural mobility of the protein (Figure 2). This analysis indicated that LptG with the G77C substitution affected the model structure through decreased amino acid mobilities near residues 199–203 and 255–265 and increased mobility near residues 204‐210.

**Figure 3:**
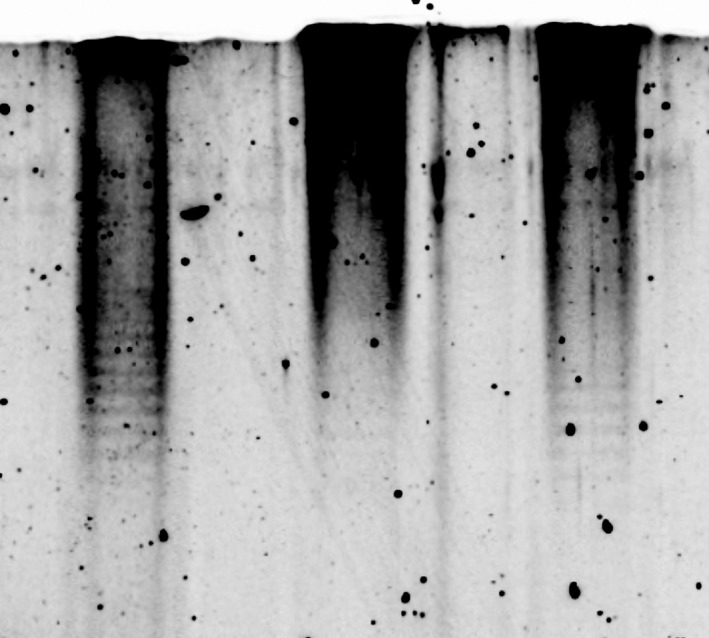
LPS profiles isolated from the clinical isolate PA34 (left) and the hypersusceptible mutants PA34‐822D (middle) and PA34‐812M (right).

**Figure 4:**
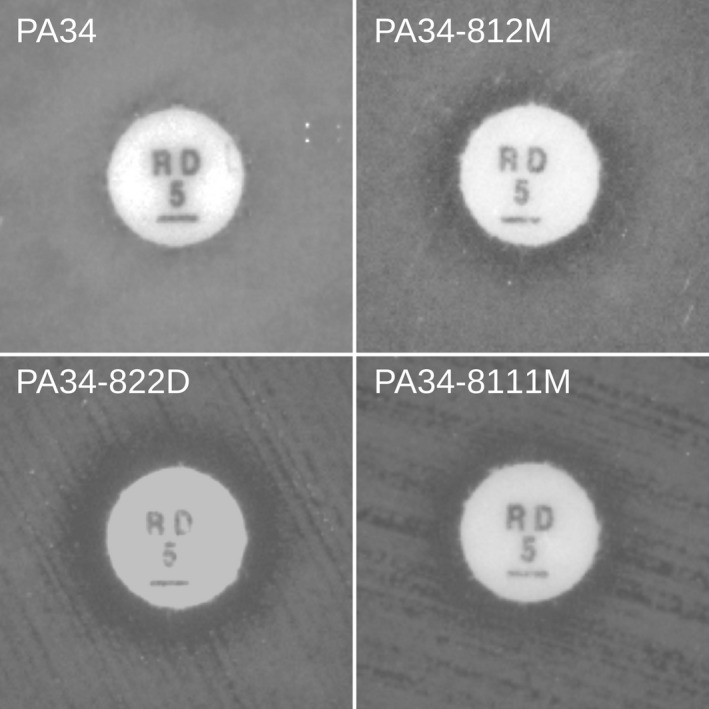
Rifampicin disk diffusion tests comparing susceptibility of PA34 against the susceptibility of three isogenic mutants, each bearing the conserved C → T mutation at bp229 in the gene encoding for the LPS transport subunit, *lptG*

To test whether the hypersusceptible isogenic mutants displayed altered LPS composition compared to the parent isolate, we evaluated LPS mobility profiles of the organisms with polyacrylamide gel electrophoresis. LPS profiles of the hypersusceptible isogenic mutants were different compared to those of the parent isolate, indicating LPS transport to the outer membrane was altered (Figure 3). LPS extractions from the hypersusceptible isogenic mutants revealed lower molecular weight LPS fragments (29–36 kDa) while extractions from the parent isolate showed both high and low molecular weight LPS fragments (29–42 kDa). LPS contributes to outer membrane permeability and *P. aeruginosa* typically has low permeability to rifampicin (Nikaido & Vaara, 1985; Vaara, 1992). Therefore, we used rifampicin to evaluate the membrane permeability of the hypersusceptible isogenic mutants. PA34 demonstrated growth up to the rifampicin disk, whereas both hypersusceptible isogenic mutants had small zones of inhibition surrounding the rifampicin disk (Figure 4). To evaluate whether permeability played a role in the hypersusceptibility of other isogenic mutants**,** an additional isolate, PA34‐8111M, was included in the analysis. PA34‐8111M had a similar susceptibility profile to PA34‐822D and PA34‐811M as evaluated by agar dilution (Table 1), being resistant to imipenem, doripenem, and meropenem, but susceptible to non‐carbapenem antibiotics. The rifampicin disk diffusion showed a similar zone of inhibition (Figure 4)**,** indicating membrane permeability as the mechanism of hypersusceptibility. Interestingly, PA34‐8111M also had the same substitution in LptG (G77C variant) as PA34‐812M and PA34‐822D.

Eight additional clinical isolates of *P. aeruginosa* with similar susceptibility profiles to the hypersusceptible isogenic mutants (i.e., susceptible to non‐carbapenem and resistant to carbapenem antibiotics) were evaluated for mutations in *lptG* and *lptF* genes using Sanger sequencing (Table 4). None of the clinical isolates showed the precise SNP responsible for the G77C substitution in the panel of clinical isolates. However, five of the eight isolates showed mutations resulting in amino acid substitutions in the LptG subunit and one showed multiple mutations resulting in amino acid substitutions in the LptF subunit (Table 5). These amino acid substitutions in the LptFG concurrent with antibiotic hypersusceptibility in both the laboratory derived mutants and clinical isolates highlight the importance of these components in the LPS transport pathway with regard to antibiotic resistance.

**Table 4:** Antibiotic susceptibility of carbapenem‐resistant *Pseudomonas aeruginosa* clinical isolates with hypersusceptible phenotype

Isolate	MIC (µg/ml)^a^					
	IPM	MER	GEN	FEP	CIP	P/T
PS423	8	1	≤1	≤1	1	≤4
PS424	≥16	≥16	≤1	8	1	8
PS425	8	0.5	≤1	≤1	1	≤4
PS426	≥16	0.5	≤1	≤1	1	≤4
PS428	≥16	8	≤1	2	≤0.25	8
PS429	≥16	2	≤1	≤1	1	≤4
PS431	≥16	4	≤1	2	0.5	8
PS432	8	0.25	≤1	≤1	0.5	≤4

Note

Susceptibilities determined by the Vitek2.

a Abbreviations: P/T, piperacillin/tazobactam; FEP, cefepime; IPM, imipenem; MEM, meropenem; GEN, gentamicin; CIP, ciprofloxacin

**Table 5:** Lpt pathway amino acid substitutions identified in *Pseudomonas aeruginosa* clinical isolate panel

Isolate	Protein	Amino acid substitutions
PS423	LptG	W123C
PS424	LptF	Q61P, G77R, Q95H, L112Q, A307P
PS425	LptG	R175H
PS426	LptG	R175H
PS428	LptG	R175H, TDH(195‐197)NGQ, L207F, HPREKRS‐EVVKLPTER(209‐225)YMKEVKHTVVMNSLTEP, A227V, D258G
PS429	LptG	R175H, TDH(195‐197)NGQ, L207F, HPREKRS‐EVVKLPTER(209‐225)YMKEVKHTVVMNSLTEP, A227V, D258G

## 4 DISCUSSION

Several publications have explored the hypersusceptible phenotype in *P. aeruginosa* (Linares et al., 2010; Mulet et al., 2011; Wolter et al., 2009). These papers have identified target pathways and transcription factors to be investigated but few gave insight into which effector genes contribute to this phenotype. Identifying candidate genes that contribute to antibiotic hypersusceptibility enables targeted research to develop novel therapeutics and restore the potency of current antibiotics to which strains of *P. aeruginosa* have become resistant.

**Figure 5:**
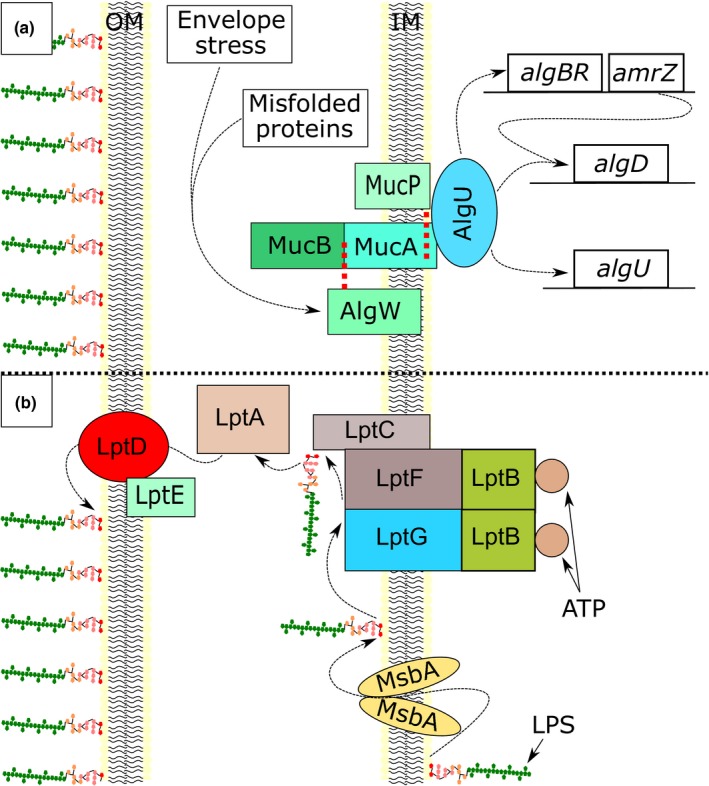
Diagram of the physiological role of AlgU and LptG. a, Environmental stressors activate AlgW to cleave and release MucB. The loss of MucB activates MucP to cleave MucA and release the sigma factor AlgU into the cytosol, allowing transcription of alginate biosynthesis products. b, LPS is transported to the outer leaflet of the inner membrane by MsbA before being transferred to the LptB_2_FG complex. LPS is then shuttled to LptDE in the outer membrane through LptC and LptA

Whole genome sequencing analysis revealed mutations in two genes that indirectly mediate entry of the antibiotic into *P. aeruginosa*, *algU* and *lptG*; *algU* through regulation of biofilm production and *lptG* through the transport of lipopolysaccharide to the outer membrane (Figure 5). The observed AlgU Q86H substitution in the hypersusceptible isogenic mutants resides in region 2 of the sigma factor, a location responsible for recognizing the −10 promoter element and binding to the RNA polymerase core. Conversion from a mucoid to a non‐mucoid phenotype has been shown to result from mutations in *algU*, consistent with the phenotype observed in the hypersusceptible mutants (Damron, Qiu, & Yu, 2009; DeVries & Ohman, 1994).

A similar study has been performed evaluating the emergence of mutations in PAO1 in response to exposure to a panel of antibiotics (Cabot et al., 2016). The selective pressure of meropenem that selected the isogenic mutants in this study and mutations observed in the PAO1 mutants favored loss‐of‐function mutations in *oprD*. However, meropenem selected for different mutations in addition to large deletions within the PAO1 genome (Cabot et al., 2016). These mutations or deletions observed in PAO1 however, did not affect the Lpt transport pathway, but did result in hypersusceptibility to colistin which was associated with a mutation in *galU*.

The use of rifampicin disk diffusion assays indicated that outer membrane permeability for the PA34 mutants was modified. Permeabilization of the outer membrane affects the retention of molecules located in the periplasm. A previous study compared extracellular AmpC activity from cultures of *P. aeruginosa* with alterations in outer membrane composition (Mulet et al., 2011). The potential leakage of AmpC is consistent with the non‐carbapenem β‐lactam hypersusceptible phenotype observed in all three isogenic mutants evaluated in this study.

Identical mutations were observed in the *lptG* gene of the hypersusceptible isogenic mutants in response to exposure to subinhibitory concentrations of carbapenem antibiotics, suggesting the importance of this gene target. The substitution G77C in LptG occurs five residues upstream of the recently identified LptB binding site in LptG (Luo et al., 2017). The substitution of this flexible glycine residue for the more rigid cysteine residue alters the protein structure. This substitution has the potential to modify the positioning of the LptB binding site in the cytoplasm or influence LptG conformational changes induced by LptB upon binding the LptFG heterodimer. While the LptG G77C substitution was not present in the panel of clinical isolates from Saudi Arabia, six of the eight had amino acid substitutions in one of the components of the LptFG heterodimer.

Previous studies have shown that disruption of LPS precursor molecule biosynthesis in the prototypic strain PAO1 results in hypersusceptibility to aminoglycosides, β‐lactams, fluoroquinolones, and rifampicin through transposon‐mediated gene disruption (Ramos‐Aires et al., 2004). Further studies have shown that disruption of the Lpt‐mediated LPS transport pathway through directed deletion of *lptD* results in hypersusceptibility to multiple classes of antibiotics (Balibar & Grabowicz, 2015). This study demonstrates how exposure to subinhibitory concentrations of carbapenem antibiotics can select for mutations in the Lpt transport pathway component *lptG*, which results in emergence of multidrug hypersusceptibility in a *P. aeruginosa* clinical isolate. The conserved G77C substitution in LptG found in multiple hypersusceptible isogenic mutants indicates one potential mechanism by which clinical isolates are able to alter outer membrane permeability and develop antibiotic hypersusceptibility in response to antibiotic exposure. In addition, this pathway was also targeted in clinical isolates of *P. aeruginosa*. It is possible that using a combination therapy consisting of a carbapenem and another anti‐pseudomonal antibiotic to treat *P. aeruginosa* could be beneficial in preventing the emergence of carbapenem resistance.

## CONFLICT OF INTERESTS

The authors declare no conflict of interest.

## AUTHOR CONTRIBUTIONS

LH assembled genomes and identified SNPs, generated protein models and mobility analysis, performed LPS profiles and rifampicin disk susceptibilities, and contributed to experimental design. RF generated and characterized isogenic mutants, performed outer membrane protein, gene expression and PFGE analyses, and contributed to experimental design. BA provided the carbapenem‐resistant clinical isolates with a hypersusceptible phenotype. AS generated sequences for genome assembly and strain selection. NH acquired strains and funding, coordinated research and contributed to experimental design.

## ETHICS STATEMENT

None required.

## Data Availability

All data are provided in the results of the manuscript with the exception of pulsed‐field electrophoresis gels and the outer membrane protein assay showing the loss of OprD, which will be made available upon request. Draft genome sequence for PA34 is located at https://www.ncbi.nlm.nih.gov/nuccore/ under the accession number PDLR00000000.
